# Fine particulate matter, suicide, and urbanicity in South Korea: A nationwide time-stratified cases-crossover study

**DOI:** 10.1016/j.isci.2025.112506

**Published:** 2025-04-22

**Authors:** RyangHa Kim, Jieun Oh, Harin Min, Seoyeong Ahn, Yejin Kim, Ayoung Kim, Cinoo Kang, Dohoon Kwon, Jinah Park, Ho Kim, Yoonhee Kim, Whanhee Lee

**Affiliations:** 1 Graduate School of Data Science, KAIST, Daejeon, South Korea; 2 Department of Public Health Sciences, Graduate School of Public Health, Seoul National University, Seoul, South Korea; 3 School of Biomedical Convergence Engineering, Pusan National University, Yangsan, South Korea; 4 Department of Information Convergence Engineering, Pusan National University, Yangsan, South Korea; 5 Department of Global Environmental Health, Graduate School of Medicine, The University of Tokyo, Tokyo, Japan; 6 Research and Management Center for Health Risk of Particulate Matter, Seoul, South Korea

**Keywords:** Public health, Environmental science

## Abstract

Previous studies reported a link between exposure to ambient fine particulate matter (PM_2.5_) and suicide. However, due to the lack of data from unmonitored areas, it has been difficult to assess heterogeneous impacts of PM_2.5_ by urbanicity. This case-crossover study investigated the relationship between short-term PM_2.5_ and suicide (2015–2019). In the overall population (65,634 suicide deaths), PM_2.5_ was marginally associated with suicide risk (odds ratio [OR]: 1.008, 95% confidence interval [CI]: 0.997–1.020). However, the association was stronger in rural areas (OR: 1.044, 95% CI: 0.996–1.095) and individuals aged 0–44 years (OR: 1.025, 95% CI: 1.002–1.048) compared to metropolitan/urban areas and older age groups. Metropolitan women aged 45–64 years (OR: 1.067, 95% CI: 1.013–1.124) and rural men aged 0–44 years (OR: 1.129, 95% CI: 0.988–1.289) showed the highest OR estimates than other subpopulations. These findings provide evidence to support more targeted suicide intervention strategies.

## Highlights

• We examined the association between short-term PM_2.5_ and suicide in South Korea
• The risk of PM_2.5_ more was pronounced in females and rural areas than other groups
• People aged 0–44 years showed the highest PM2.5 risk on suicide compared to older groups
• The higher risk in females was more evident in Metropolitan

## Graphical abstract

**Figure:**
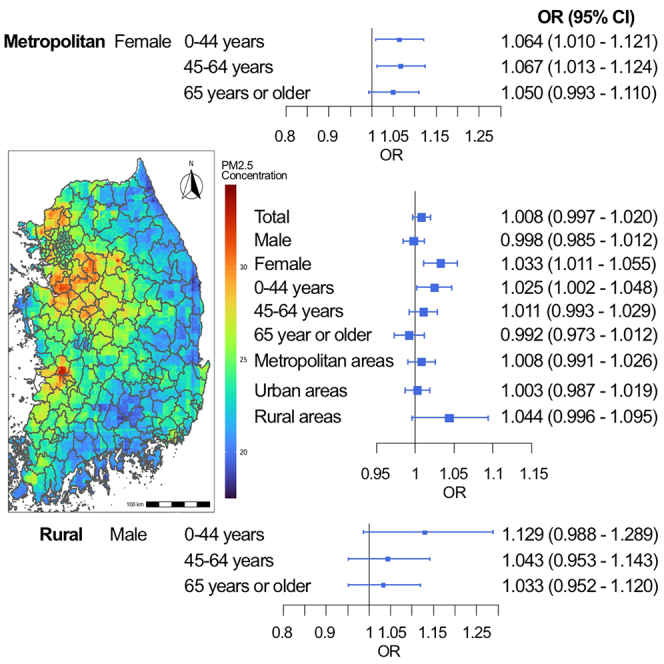

## Introduction

Suicide is a major and growing public health concern worldwide.^1^ According to the World Health Organization (WHO) report in 2019, approximately 700,000 people die by suicide each year, a number higher than deaths from breast cancer, malaria, and HIV/AIDS.^1^^,^^2^ Suicide is a leading cause of premature death, particularly the fourth leading cause of death among individuals aged 15–29 years.^3^ Suicide is also a serious social and public health issue in South Korea.^4^ An Organization for Economic Co-operation and Development (OECD) report indicated that South Korea had the highest suicide rate among OECD countries; in 2020, the suicide rate in South Korea was 24.1 per 100,000 people.^5^

Urbanicity is a multifaceted concept that influences human lifestyles, demographics, and social structures.^6^ Urbanization, which refers to changes in the size and density of cities, is a global phenomenon expected to continue for decades in both industrialized and less-industrialized countries (OECD 2006). The urbanization continuously affects social and physical environments, as well as access to health and social services.^7^ Notably, existing studies have reported that characteristics of urbanicity, such as population density, access to infrastructure, the prevalence of mental health disorders, availability of medical and leisure services, and the percentage of individuals aged 65 or older receiving basic pensions, are associated with suicide rates, particularly urban-rural disparities in suicide mortality.^8^^,^^9^

Among the various urbanicity-related risk factors for suicide, previous studies have consistently shown that exposure to outdoor fine particulate matter (PM_2.5_) is associated with a higher risk of suicide.^10^^,^^11^^,^^12^^,^^13^ Although the mechanisms are not fully understood, it has been hypothesized that exposure to PM_2.5_ may contribute to or exacerbate neurological and mental disorders associated with cognitive responses, impulsiveness, anxiety, and depression. This hypothesis has been supported by both epidemiological^14^^,^^15^ and laboratory studies.^16^^,^^17^ Specifically, increases in oxidative stress, cytokines that trigger inflammatory responses, and dysfunction of the hypothalamic-pituitary-adrenal axis due to PM_2.5_ exposure have been suggested as plausible mechanisms linking PM_2.5_ exposure to suicide.^10^^,^^18^

However, several significant knowledge gaps remain in the literature. First, because air pollution monitoring stations are primarily located in metropolitan or industrial areas, previous studies relying on monitored PM_2.5_ data have limitations in assessing the differential urban-rural risks of PM_2.5_ on suicide.^19^ The limited number of monitoring stations has resulted in the underrepresentation of rural areas, which may have different PM_2.5_-suicide relationships due to population differences,^20^ socioeconomic status (e.g., lower socioeconomic status),^21^ exposure patterns (e.g., higher daytime exposure to PM_2.5_ due to more outdoor occupations such as agriculture and fishing), and chemical compositions of PM_2.5._^22^ Additionally, risk estimates based on selected study areas could lead to biases (e.g., selection bias) when assessing nationwide population-representative risk estimates. Furthermore, due to limited data, previous studies have faced challenges in evaluating different PM_2.5_-related suicide risks by age group and sex, which can modify the risks. Age- and sex-specific analyses in the context of urbanicity, in particular, can provide valuable insights into high-risk groups across different urbanicity levels, which can inform more precise mitigation policies and resource allocations.

To address these knowledge gaps, we conducted a nationwide time-stratified case-crossover study using national mortality records from 2015 through 2019 in South Korea, employing a highly accurate machine-learning ensemble prediction model for PM_2.5_ (with a test R^2^ > 0.94) that covers all inland districts in South Korea. In this study, we aimed to examine (1) the heterogeneous association between short-term exposure to PM_2.5_ and suicide mortality depending on urbanicity level and (2) different high-risk populations by urbanicity, sex, and age through three-phase stratification analyses.

## Results

### Descriptive statistics on suicide mortality and modeled PM_2.5_ data

**Figure 1:**
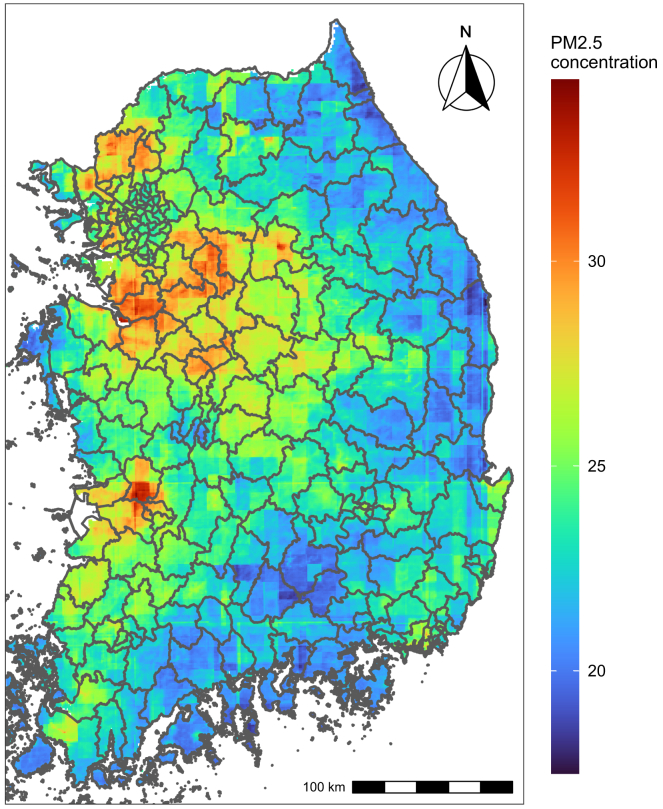
Spatial distributions of the annual averages of daily average PM_2.5_ (μg/m^3^) in South Korea from 2015 through 2019

Table 1 presents descriptive statistics for suicide deaths during the study period. A total of 65,634 suicide cases from 2015 to 2019 were included. Men (71%) and individuals under 65 years of age (0–44 years: 25%, 45–64 years: 39.9%) experienced higher suicide rates compared to women and those aged 65 years or older. Despite this, the baseline suicide risk (i.e., deaths per 100,000 persons) was highest among individuals aged 65 or older (356.2 deaths per 100,000 persons) compared to other age groups. Additionally, suicide counts and baseline risks are varied by urbanicity. The number of suicides was higher in metropolitan (43.3%) and urban areas (47.4%) compared to that in rural areas. However, the baseline suicide risk was greater in rural areas (163.3 deaths per 100,000 persons) than in metropolitan (110.7) and urban (91.2) areas. Figure 1 illustrates the geographical distribution of average PM_2.5_ concentrations during the study period (25.04 μg/m^3^ on average, with a standard deviation of 13.68 μg/m^3^ across all districts), which varied by urbanicity: 24.19 μg/m^3^ in metropolitan, 25.50 μg/m^3^ in urban, and 23.43 μg/m^3^ in rural areas. Table S1 presents the average PM_2.5_ concentrations between case and control days during the study period, and the difference is the largest in rural areas (0.37 μg/m^3^).

**Table 1:** Descriptive statistics on suicide mortality data of this study (2015–2019)

	Number of suicides	(%)	Suicide cases per 100,000 persons^a^
Total	65,634	100.0	133.2
Male	46,639	71.1	189.5
Female	18,995	28.9	77.0
0-44 years	16,436	25.0	59.3
45-64 years	26,194	39.9	176.1
65 years or older	23,004	35.0	356.2
Metropolitan areas	28,423	43.3	110.7
Urban areas	31,140	47.4	91.2
Rural areas	6,071	9.2	163.2

Metropolitan areas (above 66.7^th^ percentile of population density), urban areas (between 33.3^rd^ and 66.7^th^ percentiles of population density), and rural areas (below 33.3^rd^ percentile of population density).

a Five-year cumulative suicide cases per 100,000 persons.

### Associations between PM_2.5_ and suicide

**Figure 2:**
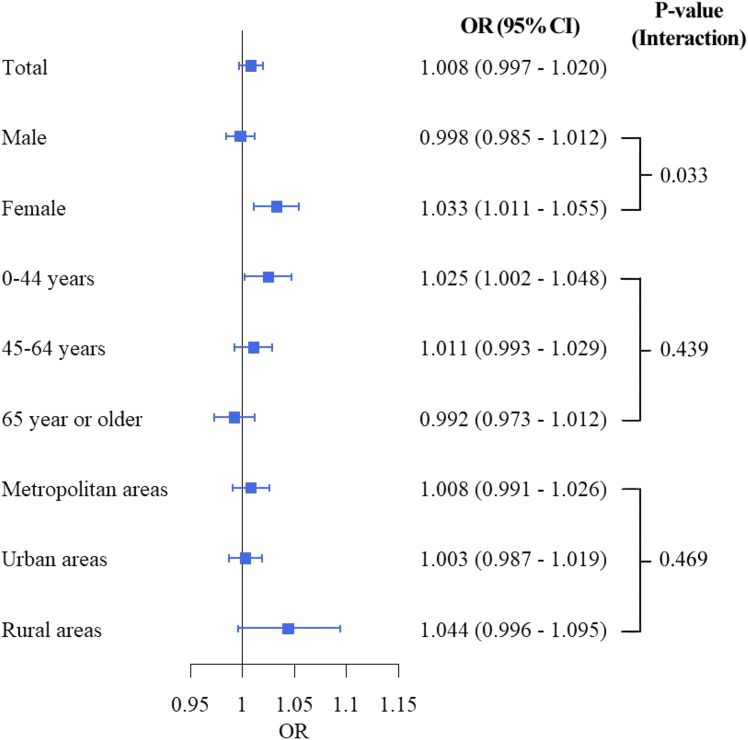
Association between PM_2.5_ and suicide mortality in the total population and by sub-populations Metropolitan areas (above 66.7^th^ percentile of population density), Urban areas (between 33.3^rd^ and 66.7^th^ percentiles of population density), and Rural areas (below 33.3^rd^ percentile of population density). *p* values were calculated by models with an interaction term between PM_2.5_ and the corresponding sub-population variable. Data are represented as point estimates (ORs; odd ratios) +/− 95% confidence intervals.

Figure 2 shows the associations between PM_2.5_ exposure and suicide mortality across the total population and within sub-populations. In the total population, short-term (lag 0–3) PM_2.5_ exposure was marginally associated with increased suicide risk, with an odds ratio [OR] of 1.008 (95% confidence interval [CI]: 0.997–1.020). This association was stronger in women (OR: 1.033, 95% CI: 1.011–1.055) than in men (OR: 0.998, 95% CI: 0.985–1.012), with a *p* value of 0.033. The association displayed a decreasing trend by age: individuals aged 0–44 years had the strongest PM_2.5_ risk (OR: 1.025, 95% CI: 1.002–1.048), compared to those aged 65 years or older (OR: 0.992, 95% CI: 0.973–1.012), although the statistical evidence was weak (*p* value: 0.439). Moreover, based on the point estimates, rural areas exhibited a higher association between PM_2.5_ and suicide risk (OR: 1.044, 95% CI: 0.996–1.095) compared to metropolitan (OR: 1.008, 95% CI: 0.991–1.026) and urban areas (OR: 1.003, 95% CI: 0.987–1.019).

### Double-phase stratified associations between PM_2.5_ and suicide

**Figure 3:**
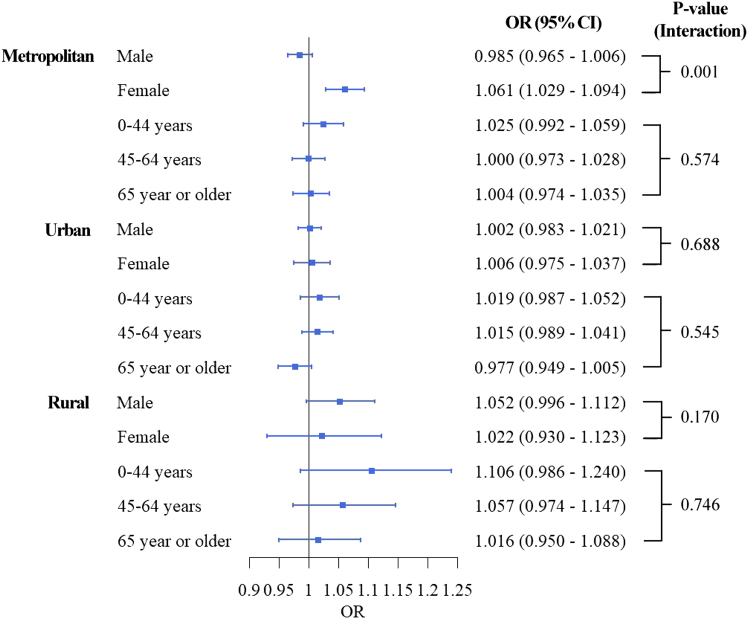
Age or Sex-specific association between PM_2.5_ and suicide mortality by urbanicity Metropolitan areas (above 66.7^th^ percentile of population density), Urban areas (between 33.3^rd^ and 66.7^th^ percentiles of population density), and Rural areas (below 33.3^rd^ percentile of population density). *p* values were calculated by models with an interaction term between PM_2.5_ and the corresponding sub-population variable. Data are represented as point estimates (ORs; odd ratios) +/− 95% confidence intervals.

Figure 3 presents results from the double-stratification analyses by urbanicity and age/sex. The age group- and sex-specific associations between PM_2.5_ and suicide risk varied by urbanicity. In metropolitan areas, the difference in PM_2.5_ risks between sexes (higher risk in women) observed in the total population (Figure 2) was evident (*p* value <0.001). However, this pattern diminished in urban areas and was reversed in rural areas, where men exhibited a higher PM_2.5_ risk (OR: 1.052, 95% CI: 0.996–1.112), although the statistical evidence was weak (Urban *p* value: 0.688, rural *p* value: 0.170). The weak decreasing pattern by age observed in the total population (Figure 2) was also detected in the double-stratification analyses, although the absolute risk size varied by urbanicity. The age-specific association between PM_2.5_ and suicide risk was not statistically significant in metropolitan and urban areas. In rural areas, individuals aged 0–44 years had the highest risk (OR: 1.106, 95% CI: 0.986–1.240) compared to individuals aged 65 years or older (OR: 1.016, 95% CI: 0.950–1.088), although the statistical evidence was weak.

### Three-phase stratified associations between PM_2.5_ and suicide

**Figure 4:**
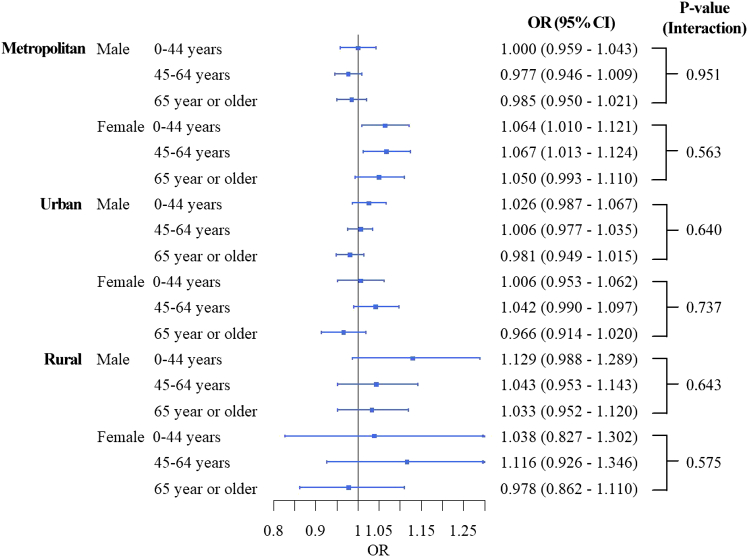
Age-sex-specific association between PM_2.5_ and suicide mortality by urbanicity Metropolitan areas (above 66.7^th^ percentile of population density), Urban areas (between 33.3^rd^ and 66.7^th^ percentiles of population density), and Rural areas (below 33.3^rd^ percentile of population density). *p* values were calculated by models with an interaction term between PM_2.5_ and the corresponding sub-population variable. Data are represented as point estimates (ORs; odd ratios) +/− 95% confidence intervals.

Figure 4 illustrates results from the triple-stratification analysis by urbanicity, age, and sex. In metropolitan areas, women aged 45–64 years showed an association between PM_2.5_ and suicide (OR: 1.067, 95% CI: 1.013–1.124), with less prominent associations in older age groups in women. In urban areas, no sub-groups exhibited a significant association between PM_2.5_ and suicide mortality. In rural areas, men aged 0–44 years had the highest PM_2.5_ risk for suicide (OR: 1.129, 95% CI: 0.988–1.289) compared to other age groups, whereas the related statistical evidence was uncertain. Women in rural areas did not show a significant relationship between PM_2.5_ and suicide mortality. Table S2 provides the counts of suicides by subgroup, corresponding to Figure 4.

### Sensitivity analysis results

Lastly, sensitivity analyses (Table S3) confirmed that our estimates and patterns by sex and age group remained consistent across different PM_2.5_ levels, temperatures, and confounder adjustments.

## Discussion

This study evaluated the association between short-term exposure to PM_2.5_ and suicide mortality risk in South Korea using nationwide mortality and PM_2.5_ data. Additionally, it investigated whether this association varied by urbanicity, sex, and age group to identify specific high-risk populations. In the total population, we found that the association between PM_2.5_ and suicide was more pronounced in women than in men. This pattern persisted in metropolitan areas, where young (0–44 years) and middle-aged women (aged 45–64 years) exhibited the highest PM_2.5_-related suicide risk compared to other sex and age groups. Specifically, men aged 0–44 years in rural areas had a marginally higher PM_2.5_-related suicide risk compared to senior men (aged 65 or older) in the same areas.

Firstly, our study found that the association between PM_2.5_ and suicide mortality risk was higher in rural areas compared to metropolitan and urban areas, based on the point estimates. This finding is consistent with recent research in the United States, which reported a higher PM_2.5_ risk in rural areas.^12^ The evidence provided by this study is somewhat limited, but several hypotheses may explain the elevated PM_2.5_-related suicide risk in rural areas. One hypothesis involves baseline differences in suicide risk. As indicated in Table 1, the baseline suicide risk (suicides per 100,000 people) was higher in rural areas than in other regions of South Korea. Similar disparities in baseline suicide risk between urban and rural areas have been reported in Germany,^8^ Finland,^23^ and a review article including a total of 24 papers.^24^ Potential factors contributing to higher baseline suicide risk in rural areas include stigmatization of individuals with mental health issues, reduced mental health services, higher levels of poverty and unemployment, and easier access to lethal means^24^—factors commonly observed in rural South Korea.^6^^,^^25^ We found that the difference in PM_2.5_ concentrations between case and control days was the largest in rural areas (Table S1). Although the relevant evidence is limited, this result implies that the short-term fluctuation of PM_2.5_ might be higher in rural areas. We cautiously conjecture that irregular PM_2.5_-related activities in rural areas, such as incineration of agricultural waste, agricultural machinery usage, and fertilizer use, etc., might affect the sudden negative biological reactions that can be linked reduced vulnerabilities to PM_2.5_-related mortality. Another factor is occupational status; rural areas may experience higher PM_2.5_ exposure due to daytime activities such as agriculture and fisheries. Furthermore, recent research in the western United States has suggested that PM_2.5_ from agricultural activities, biomass burning, and coal combustion in rural areas could be more hazardous to human health than PM_2.5_ in urban areas.^26^ In addition, a study in Canada highlighted that lower access to mental health services, high-risk alcohol consumption, and easier access to methods of suicide (e.g., hanging) contribute to higher suicide risk among rural populations,^27^ and among them, high-risk alcohol consumption and accessibility to suicide methods in rural areas could be related to higher PM_2.5_-related suicide association because PM_2.5_ also has been regarded as a “trigger” of suicide attempts.^18^ South Korea also showed generally a higher percentage of high-risk alcohol consumption in rural areas compared to other areas.^25^ These findings suggest that the higher daytime exposure to more hazardous PM_2.5_, unhealthy behaviors, and higher access to suicide methods in rural areas might strengthen the association between PM_2.5_ and suicide compared to metropolitan and urban areas, although further research is needed.

Secondly, this study revealed that the association between PM_2.5_ and suicide mortality risk is higher in women than in men, with this pattern being more pronounced in metropolitan areas compared to rural areas. Previous research has produced mixed results regarding sex differences in the association between air pollution and suicide risks. A multi-city study in Northeast Asian countries reported no significant difference by sex.^11^ Conversely, some studies have indicated that men have a higher risk associated with PM or suicide,^28^^,^^29^ while others have found a higher risk in women.^13^^,^^30^ Factors such as higher PM_2.5_ exposure due to increased economic or outdoor activities in females, the immunosuppressive effects of androgens, and the inflammatory effects of estrogen in women may contribute to these discrepancies.^10^^,^^30^ According to the “suicide survey in 2018” that has been operated by the Ministry of Health and Welfare in South Korea,^31^ suicide attempts (defined by psychiatrists) and ideation in females were more related to psychiatric symptoms and problems in social relationships compared to males. Although relevant evidence is highly limited, we conjecture that this result could be related to a rapid increase in women’s participation in economic activities in recent years (48.5% in 1995 and 53.5% in 2019).^32^ This increase may lead to higher stress, occupational PM_2.5_ exposures, and mental health issues due to inadequate time for adaptation. South Korea also has the largest salary gap by gender in the OECD, with females earning 63% of the average salaries of males (56% of women were employed versus 76% of men).^33^ Especially, the higher association between PM_2.5_ and suicide in females was observed only in young and middle-aged females residing in metropolitan areas (Figures 3 and 4), these results might support our hypothesis that sudden, higher economic activities, and resultant occupational stresses and increases in PM_2.5_ exposure in young and middle-aged females could be related to higher PM_2.5_ risk estimates in young and middle-aged females in metropolitan areas. These gender inequalities in economic activities and incomes could be related to higher stress and mental malfunctioning in females. Moreover, historically, Korean (occupational) culture has been substantially patriarchal,^34^ and increases in stress due to economic activities and household work that concurrently have been given to working females, which can be a critical trigger of suicide attempts, have been recognized as a severe and growing social issue in South Korea.^35^ Although further research with more quantitative evidence is needed, our findings suggest that tailored action plans for suicide prevention and enhanced mental health support for women in metropolitan areas are warranted.

Another important finding of this study is that men in rural areas, particularly younger men, show a higher risk of suicide associated with PM_2.5_ exposure. Related to suicide attempts, males in South Korea were generally more sensitive to income loss and diseases, compared to females.^31^ In South Korea, based on Life Expectancy Statistics 2024, the general health status of males has been lower than that of females.^36^ We also propose that the specific characteristics of rural men in South Korea should be considered when interpreting their higher PM_2.5_-related suicide risk. Unlike findings in Australia, where older rural men were identified as high-risk,^20^ younger rural men (aged 0–44 years) exhibit the highest risk in South Korea. This discrepancy may be attributed to unique rural economic conditions in South Korea. First, rural areas have shown lower economic activities and poorer socioeconomic status than urban or metropolitan areas in South Korea.^6^^,^^25^ Further, as we mentioned earlier, males have been regarded as major members of economic activity in South Korea,^34^ thus rural young or middle-aged males could get more stress in finding secure or high-income job opportunities than those in urban or metropolitan areas with more economic opportunities. Moreover, in recent years, many urban middle-aged individuals, particularly “middle-aged men,” have attempted to “return to rural areas” to start new businesses, especially in agriculture.^37^ However, many of these ventures have failed, with business failures and lower-than-expected incomes identified as major issues, alongside conflicts with neighbors, limited social amenities, and inadequate medical and transport facilities.^38^ Given that men are particularly sensitive to income loss, the stress and mental health decline associated with failed rural business ventures may increase the roles of PM_2.5_ as a “trigger” of suicide.^18^ However, due to limited data availability, this study could not analyze the motivations or causes of suicide in detail. Given the severe social implications of high suicide rates in rural areas, further research with more comprehensive datasets is necessary to test this hypothesis.

This study has several strengths. Firstly, to our knowledge, it is the first nationwide investigation assessing the association between short-term PM_2.5_ exposure and suicide mortality using population-representative mortality and air pollution data in South Korea. This approach likely reduces selection and measurement biases compared to previous studies focusing on selected monitored areas or larger administrative units than the “district” used in this study. Additionally, by examining the heterogeneous associations based on urbanicity and age-sex-specific analyses, we identified high-risk populations according to urbanicity level: middle-aged women (45–64 years) in metropolitan areas and younger men (0–44 years) in rural areas. A recent Chinese nationwide study also showed that an increase in short-term PM_2.5_ might raise suicide risks. However, unlike our results, this study exhibited that women over 65 years had a higher vulnerability to PM_2.5_ and suicide relationships.^39^ Moreover, this study estimated the reducible numbers of suicide by the compliance of the Air Pollution Action Plan of China. Future studies in South Korea should examine the reasons for the differences in high-risk populations and show the potential benefits of following the South Korean air quality standards (daily PM2.5 < 35 μg/m^3^) or the WHO air quality guidelines (daily PM_2.5_ < 25 μg/m^3^).

### Limitations of the study

Nonetheless, this study has several limitations. Firstly, despite employing a time-stratified case-crossover design to control for time-invariant confounders, unmeasured time-dependent confounders such as daily mood, activities, and high-risk drinking could have influenced our estimates. Secondly, due to an insufficient number of cases, we were unable to examine more specific age (for individuals under 44 years) or urbanicity categories, which limited our ability to produce robust statistical estimates. In other words, the current study results should be demonstrated carefully in further studies, including sufficient samples. Thirdly, we used district-average PM_2.5_ concentrations as a proxy for individual exposures because the national mortality data only provided district-level addresses (with a median district size of 397 km^2^, approximately 1.7 times larger than the median size of US ZIP code areas). As a result, potential exposure misclassifications and measurement errors may exist. In particular, in general, rural districts have larger area sizes than urban or metropolitan areas in South Korea, thus the potential biases due to misclassifications and measurement errors could be higher in rural districts. This bias could be reduced if more specific addresses were available; however, the current related legislation (e.g., Act of the Protection of Personal Information) does not allow the use or opening of more specific residential addresses rather than “district-level” addresses when researchers used the mortality data provided by Statistics Korea. Further, due to the limited data availability, this study could only consider residential addresses of registered death cases for the exposure allocation. However, although it could be conjectured that the mobility of individuals who are at-mortality risk population (e.g., senior populations) might be not very large, it is substantially difficult to demonstrate this assumption and allocate more precise PM_2.5_ exposure based on their movements or places where case people spent more time than their registered addresses because the mortality database does not include the related records. Therefore, to improve these historical and fundamental limitations of the environmental epidemiology studies based on the registered mortality data, future studies should consider a large cohort including more precise databases including mobilities, specific addresses with high spatial resolution, and spatially and temporally matched exposures and outcomes and perform proper statistical analyses corresponding to these larger databases.

### Conclusion

We explored the nationwide association between short-term PM_2.5_ exposure and suicide mortality risk in South Korea. Through stratification analyses, we identified that middle-aged women (45–64 years) residing in metropolitan areas and younger men (0–44 years) living in rural areas may be at the highest risk regarding the association between PM_2.5_ and suicide. These findings suggest that more precise public health policies and targeted social interventions are needed, considering regional and individual characteristics to reduce suicide risks associated with PM_2.5_ exposure more effectively.

## Resource availability

### Lead contact

Requests for further information and resources should be directed to and will be fulfilled by the lead contact, Whanhee Lee (whanhee.lee@pusan.ac.kr).

### Materials availability

This study did not generate new unique materials.

### Data and code availability

• Data: Data reported in this paper will be shared by the lead or corresponding author upon request.
• Code: Analytic code (R code) will be shared by the lead or corresponding author upon request.
• Additional information: any additional information required to reanalyze the data reported in this paper is available from the lead or corresponding author upon request.

## Acknowledgments

This work was supported by Institute of Information & communications Technology Planning & Evaluation (IITP) under the Artificial Intelligence Convergence Innovation Human Resources Development (IITP-2025-RS-2023-00254177) grant funded by the Korea government (10.13039/501100014188MSIT). This work was also supported by the 10.13039/501100010700National Institute of Environmental Research (NIER) funded by the 10.13039/501100003562Ministry of Environment (MOE) of the Republic of Korea (NIER-2021-03-03-007).

## Author contributions

R.K., formal analysis, data curation, visualization, and writing – original draft; J.O., data curation and writing – review and editing; H.M., data curation and writing – review and editing; S.A., Y.K., A.K., C.K., D.K., and J.P., data curation; H.K., writing – review and editing and funding acquisition; Y.K., writing – review and editing; W.L., conceptualization, methodology, investigation, supervision, writing – original draft, and funding acquisition.

## Declaration of interests

The authors declare no competing interests.

## STAR★Methods

### Key resources table

**Table:** 

REAGENT or RESOURCE	SOURCE	IDENTIFIER
**Software and algorithms**		

R Software (4.3.3)	Free Software Foundation’s GNU General Public License	https://www.r-project.org/

### Experimental model and study participant details

This study is an epidemiological study based on publicly available mortality data collected by Statistics Korea. Thus, this study is not applicable to experimental models. In addition, since the mortality data the study used is based on the nationwide registration, there were no inclusion or exclusion criteria for the data collection (i.e., there are no restrictions for the study participation). We disclose that ethical approval was not required for this study because it used publicly available, secondary, de-identified suicide mortality data.

### Method details

#### Study population and suicide data

The study population comprised all individuals who died by suicide and were registered with Statistics Korea. We specifically obtained individual-level mortality data for all 247 districts in mainland South Korea (excluding Jeju and Ulleung islands) from January 1, 2015, to December 31, 2019. The International Classification of Diseases, 10^th^ Revision (ICD–10) was used to define deaths due to suicide (ICD-10: X60–X84) (Kim et al. 2018).

#### Urbanicity level

We first collected the annual population density (persons per km^2^) during the study period (2015–2019) from a community-level health-related factor database provided by the Korea Disease Control and Prevention Agency.^25^ Population density was selected to approximate the urbanicity level of South Korean districts. It was identified as one of the most suitable indicators of urbanicity level in the country, although different countries adopt significantly varied metrics to determine urbanicity levels and the thresholds for urban and rural areas.^6^ South Korea is one of the most densely populated countries in the OECD.^40^ Approximately 45% of the population resides in the seven largest metropolitan cities, which account for only 5% of the total land area.^6^ Furthermore, among administrative districts, the highly urbanized areas of Korea (especially Seoul and Busan, the two largest metropolitan cities) exhibit the highest population densities. Consequently, we determined that population density is the most suitable indicator of urbanization level in Korean districts.

For each district, we averaged population density across the study period and used these averages to categorize the districts into three categories based on their tertiles (Byun et al. 2024): metropolitan areas (above 66.7^th^ percentile; 3278.8 to 27068.6 persons/km^2^), urban areas (between 33.3^rd^ and 66.7^th^ percentiles; 161.0 to 3120.3 persons/km^2^), and rural areas (below 33.3^rd^ percentile; 19.7 to 158.6 persons/km^2^).

#### Air pollution and environmental data

We collected a nationwide modeled PM_2.5_ from 2015 to 2019 provided by the AiMS-CREATE team.^41^^,^^42^ Specifically, we obtained the predicted daily concentrations of ambient PM_2.5_ (24-hour average; μg/m^3^) at a 1 km^2^ spatial resolution with excellent performance (test R^2^ = 0.94) across mainland South Korea, using a machine-learning ensemble prediction model. The ensemble model incorporates three machine-learning algorithms (random forest, extreme gradient boosting, and deep neural networks) trained individually with multiple satellite-based variables. More detailed information on the PM_2.5_ prediction model is provided in previously published papers.^41^^,^^42^^,^^43^ Table S4 presents the performance of the air pollution prediction model used in this study.

The district is the smallest address level that the national mortality database could provide for this study. Therefore, daily PM_2.5_ concentration predictions at 1 km^2^ were aggregated for each district by averaging the predictions at grid cells with centroid points inside the boundary of each district. We used a district-level 4-day moving average of PM_2.5_ concentrations (lag 0-3) based on the Akaike Information Criteria (we found this moving average period with the lowest information criteria using a grid search from lag 0 to 5-day moving average).^11^^,^^13^^,^^44^

As potential confounders and covariates, we first collected district-level daily outdoor temperature (°C) and relative humidity (%) from the ERA-5 Land global reanalysis dataset, including 24-hour average 2m air temperature (K) and 2m dew point temperature (K). The ERA-5 dataset has a horizontal resolution of 0.1 ° × 0.1 °, with a native spatial resolution of 9–11 km, which was aggregated into district units by averaging the values at grid cells with centroid points inside the boundary of each district.^45^ We also obtained district-specific modeled outdoor ozone (ppm) data from 2015 to 2019, provided by the AiMS-CREATE team, based on a machine-learning ensemble model.^42^^,^^43^

### Quantification and statistical analysis

In the main analysis, we created a time-stratified case-crossover dataset for each urbanicity level, age group, and sex (i.e. combinations of the three urbanicity levels, age groups, and sex). We designated the case day as the date of suicide and the matched control days as the same day of the week within the same month of the same year. This self-matching by month and year controls for confounding variables that do not markedly change within a month, such as age, sex, body mass index, health behaviors (e.g., smoking, exercise), and other time-invariant factors, including regional indicators (e.g., medical service accessibility, demographic composition, socioeconomic environments). Matching by day of the week controlled for potential mortality differences within a week, and the bidirectional matching (before and after the case day) could reduce potential confounding by seasonality and long-term time trends.^46^ This approach has been widely used to evaluate the impacts of short-term environmental exposure on acute health outcomes.^45^^,^^47^^,^^48^

For each case-crossover dataset, we fitted a conditional logistic regression model to estimate the association between short-term exposure to PM_2.5_ (lag 0–3) and the risk of suicide mortality. Modeled ozone (lag 0–1), dewpoint temperature (lag 0–1), and temperature were adjusted in the model as time-varying confounders. For temperature adjustment, the distributed lag nonlinear model was used to capture nonlinear and delayed roles of temperatures.^49^ The exposure-response function was modeled using a natural cubic spline function with two internal knots at the 25^th^, 50^th^, and 75^th^ percentiles of the temperature distribution. The lag-response function was modeled with a natural cubic spline with an intercept and one internal knot (at lag day 1). The selection of exposure-lag-response relationships between temperature and suicide has been widely used and validated in previous studies on short-term environmental stressors and suicide mortality.^11^^,^^13^^,^^50^ We calculated the odds ratio (OR) for a 10μg/m^3^ increase in PM_2.5_ to measure the association between short-term exposure to PM_2.5_ and suicide mortality. Thus, if OR is larger/smaller than 1, it indicates that an increase in PM_2.5_ might be associated with an increased/decreased risk of suicide mortality, respectively.

In addition, to avoid the confusion of interpretation, we used the term “association between PM_2.5_ and suicide” to signify the “positive” (direction) association between PM_2.5_ and suicide across the manuscript because it could be expected that an increased PM_2.5_ might escalate the risk of suicide because of the toxicity of the PM_2.5_. This positive association has been widely observed in previous studies.^18^

#### Sub-group analysis

Age and sex have been identified as risk factors for suicide^4^ as well as effect modifiers of the association between PM_2.5_ and suicide mortality risk in multiple studies.^10^^,^^11^^,^^29^ First, we divided the ages (at death) into three categories: individuals aged 0-44 years, 45-64 years, and 65 years or older. The age categories were used for stratified analyses by sex and urbanicity levels (metropolitan/urban/rural areas). To find more specific high-risk populations, we conducted three-phase stratified analyses: first, we estimated the associations between PM_2.5_ and suicide mortality for each sex, age group, and urbanicity level (single-phase stratification). Second, we examined the associations for each sex and age group by urbanicity level (two-phase stratification). Lastly, we assessed the associations by sex, age group, and urbanicity level, concurrently (three-phase stratification).

To test the difference in OR estimates among subgroups, we added an interaction term in the main model for each subgroup and derived the corresponding p-value of the interaction term. In addition, we operationally defined the terms “marginally or weakly related” when the estimated p-value was lower than 0.2.

#### Sensitivity analysis

Finally, we conducted sensitivity analyses to examine whether our results were consistent across different modeling specifications regarding lag days and confounder adjustments. All statistical analyses were performed using R software (version 4.2.1) with libraries “*dlnm*” and “*survival*” were used.
