# Supplementary material for: Fine particulate matter, suicide, and urbanicity in South Korea: A nationwide time-stratified cases-crossover study
Source: iScience. 2025 Apr 22;28(5):112506. doi: 10.1016/j.isci.2025.112506 (PMC12136848; doi:10.1016/j.isci.2025.112506)
Supplement: Document S1. Tables S1–S4 [file mmc1.pdf]

iScience, Volume *28*

Supplemental information

Fine particulate matter, suicide,

and urbanicity in South Korea: A nationwide

time-stratified cases-crossover study

RyangHa Kim, Jieun Oh, Harin Min, Seoyeong Ahn, Yejin Kim, Ayoung Kim, Cinoo Kang, Dohoon Kwon, Jinah Park, Ho Kim, Yoonhee Kim, and Whanhee Lee

Table S1. Summarized $PM_{2.5}$ concentrations in metropolitan/urban/rural areas by case and control days. Values: Mean (Standard deviation). Metropolitan and urban areas showed higher concentrations compared to rural areas.

	Total areas	Metropolitan areas	Urban areas	Rural areas
**Case days**	24.81 (12.42)	24.32 (12.06)	25.50 (13.02)	23.60 (10.67)
**Control days**	24.55 (12.12)	24.13 (11.88)	25.25 (12.66)	23.23 (10.50)

Table S2. Number of suicides by urbanicity, age, and sex. Metropolitan areas (above 66.7th percentile of population density), Urban areas (between 33.3rd and 66.7th percentiles of population density), and Rural areas (below 33.3rd percentile of population density). The number of suicides was heterogeneous by urbanicity level.

			Number of suicides	%
**Metropolitan areas**	**Male**	**0-44 years**	4326	7.2
		**45-64 years**	7689	12.8
		**65 year or older**	6163	10.3
	**Female**	**0-44 years**	2620	4.4
		**45-64 years**	2760	4.6
		**65 year or older**	2360	3.9
**Urban areas**	**Male**	**0-44 years**	4690	7.8
		**45-64 years**	8574	14.3
		**65 year or older**	6957	11.6
	**Female**	**0-44 years**	2523	4.2
		**45-64 years**	2932	4.9
		**65 year or older**	2766	4.6
**Rural areas**	**Male**	**0-44 years**	636	1.1
		**45-64 years**	1567	2.6
		**65 year or older**	1901	3.2
	**Female**	**0-44 years**	235	0.4
		**45-64 years**	376	0.6
		**65 year or older**	794	1.3

Table S3. Sensitivity analysis. Numbers: odd ratios per 10 μg/m^3 of $PM_{2.5}$. The sensitivity analysis results showed our main results might be robust.

	Main	$PM_{2.5}$ lag 0-1	$PM_{2.5}$ lag 0-2	Temperate lag 0-3	Temperate lag 0-6	Without O_3 adjustment	Without dewpoint temperature adjustment
**Total**	1.008 (0.997 - 1.020)	1.005 (0.995 - 1.016)	1.005 (0.994 - 1.016)	1.007 (0.996 - 1.019)	1.009 (0.997 - 1.021)	1.006 (0.996 - 1.017)	1.006 (0.995 - 1.018)
**Male**	0.998 (0.985 - 1.012)	0.997 (0.985 - 1.010)	0.996 (0.983 - 1.009)	0.997 (0.984 - 1.011)	0.999 (0.985 - 1.013)	0.997 (0.984 - 1.010)	0.997 (0.984 - 1.010)
**Female**	1.033 (1.011 - 1.055)	1.025 (1.006 - 1.045)	1.029 (1.008 - 1.049)	1.032 (1.010 - 1.054)	1.033 (1.011 - 1.056)	1.030 (1.010 - 1.051)	1.029 (1.008 - 1.051)
**0-44 years**	1.025 (1.002 - 1.048)	1.015 (0.995 - 1.036)	1.022 (1.000 - 1.044)	1.024 (1.002 - 1.047)	1.024 (1.000 - 1.047)	1.025 (1.004 - 1.047)	1.023 (1.001 - 1.045)
**45-64 years**	1.011 (0.993 - 1.029)	1.010 (0.993 - 1.026)	1.010 (0.992 - 1.027)	1.010 (0.992 - 1.028)	1.015 (0.996 - 1.034)	1.007 (0.990 - 1.024)	1.008 (0.990 - 1.026)
**65 year or older**	0.992 (0.973 - 1.012)	0.992 (0.975 - 1.010)	0.988 (0.969 - 1.007)	0.992 (0.972 - 1.011)	0.991 (0.970 - 1.011)	0.992 (0.974 - 1.011)	0.992 (0.973 - 1.011)
**Metropolitan areas**	1.008 (0.991 - 1.026)	1.006 (0.991 - 1.022)	1.005 (0.988 - 1.021)	1.007 (0.990 - 1.025)	1.006 (0.988 - 1.024)	1.003 (0.987 - 1.020)	1.004 (0.987 - 1.021)
**Urban areas**	1.003 (0.987 - 1.019)	0.996 (0.982 - 1.011)	0.999 (0.984 - 1.015)	1.002 (0.986 - 1.019)	1.006 (0.990 - 1.024)	1.004 (0.989 - 1.020)	1.005 (0.989 - 1.021)
**Rural areas**	1.044 (0.996 - 1.095)	1.062 (1.017 - 1.109)	1.059 (1.012 - 1.108)	1.043 (0.994 - 1.093)	1.042 (0.992 - 1.094)	1.041 (0.996 - 1.088)	1.037 (0.990 - 1.086

Table S4. Summary statistics on the performance of the $PM_{2.5}$ and ozone prediction models during the study period (2015 to 2019). RMSE: root mean squared error, MAE: mean absolute error. RMSE and MAE are in the unit $PM_{2.5}$ of μg/m^3 and ozone (ppm). The prediction models cover all inland areas in South Korea with 1km^2 spatial resolution.

		R^2	RMSE	MAE
**$PM_{2.5}$**	**Total years**	0.944	3.219	2.187
	**2015**	0.889	3.375	2.179
	**2016**	0.882	3.352	2.290
	**2017**	0.925	3.283	2.258
	**2018**	0.957	3.453	2.148
	**2019**	0.965	3.135	2.138
**Ozone**	**Total years**	0.944	0.004	0.002
	**2015**	0.889	0.004	0.002
	**2016**	0.882	0.004	0.002
	**2017**	0.925	0.004	0.002
	**2018**	0.957	0.004	0.002
	**2019**	0.965	0.004	0.002
